# Human, Animal and Automata Attributions: an Investigation of the Multidimensionality of the Ontologization Process

**DOI:** 10.1007/s42087-022-00277-8

**Published:** 2022-03-31

**Authors:** Monica Pivetti, Giannino Melotti, Anna Maria Meneghini, Elisa Puntin, Paola Villano

**Affiliations:** 1 Department of Human and Social Sciences, University of Bergamo, Bergamo, Italy; 2 Department of Education Studies “Giovanni Maria Bertin”, University of Bologna, Bologna, Italy; 3 Department of Human Sciences, University of Verona, Verona, Italy

**Keywords:** Prejudice, Ontologization approach, Dehumanization, Chinese people, Roma people, Human attribution

## Abstract

The ontologization process involves the use of social representation relating to the human–animal binary to classify ingroup and outgroup members. To date, no study has investigated the multidimensional nature (i.e. human, animal and automata) of the ontologizing process via structural equation modelling (SEM). Four hundred and twenty-one Italian participants were asked to attribute 24 positive/negative, human/animal/automata associates to each of three target groups: typical Roma/Chinese/Italian. Results showed that the proposed six-factor model (i.e. positive/negative, human/animal/automata essence) was statistically robust for each of the three groups. The Roma group was animalized by attributing more animal negative associates than any other target group, whereas the Chinese group was mainly given a robot positive essence.

## Introduction

Discrimination, prejudice and xenophobia persist in our society, even though they are condemned by legal and social norms (e.g. Ceobanu & Esandell, 2010). Prejudice has been considered to constitute the emotional component of attitudes towards social groups and has been traditionally defined as reflecting overt intergroup hostility, in particular towards marginalized groups (Allport, 1954; Brown, 2011; Dovidio & Jones, 2019).

In intergroup relations, outgroup derogation may lead to an expression of subtle prejudice, thereby protecting the ingroup from being seen as transgressors of anti-racist norms, or it may lead to an expression of openly xenophobic attitudes and behaviours. For instance, an incident recently occurred in an Italian supermarket in which a customer, while she was in the checkout queue waiting to pay, addressed the cashier of Dominican origin, who had asked her to stand a little further back to comply with the social distancing required by the COVID-19 rules, with the following words: “Shut up and just do your job, monkey face. If you don’t like where I am, go back to your country” (Livorno Today, February 12th 2021). The racist insult of the supermarket customer can be seen as an example of dehumanization, i.e. the denial of the full presence of the human essence, to a member of an outgroup, the cashier, who is considered to belong to the outgroup because of the colour of her skin (being Dominican).

From a socio-psychological perspective, the study of dehumanization has received considerable attention over the last 10 years and has gained strong empirical support (e.g. Haslam & Loughnan, 2014; Vaes et al., 2012). Dehumanizing means perceiving a target as belonging to a lower order of humanity (Haslam, 2006; Leyens et al., 2000). It emerges when the target is perceived as not sharing normative values with the ingroup (see Schwartz & Struch, 1989). While humanity is strongly associated with ingroups (e.g. Bain et al., 2009), dehumanization is the process by which outgroup members are discriminated against by being perceived as less than human (i.e. attribute-based dehumanization) or by being associated with a more animal-like or automata-like status (i.e. metaphor-based dehumanization) (see Loughnan et al., 2009).

Haslam (2006) proposed a comprehensive “dehumanization model” in which humanness is defined by attributes that are unique to humans (human uniqueness (HU)) and those that are essential to being human (human nature (HN)). For example, some HU attributes are higher cognition, moral sensibility and sophistication, while HN attributes are emotionality, interpersonal warmth, flexibility and animation. In an intergroup context, the denial of HU attributes (e.g. moral sensibility) may lead to members of the ingroup likening outgroup members to animals, whereas a denial of HN attributes (e.g. emotionality) may lead them to liken outgroup members to automata (Bain et al., 2009; Haslam et al., 2008). With regard to animalistic dehumanization, some empirical studies (e.g. Viki et al., 2006) have found that the participants were prone to associating their ingroup more with human- vs. animal-related words in comparison to outgroups. In the case of mechanistic dehumanization, HN attributes are denied, and other people (outgroup members) are represented as unfeeling, cold, passive, rigid and lacking individuality. Martínez et al. (2012), for instance, found that the Spanish participants in their study linked Roma surnames more with animal-related words and German surnames with machine-related words.

Alongside the approach focused on dehumanization, a strand of research on ethnic prejudice is that of ontologization process (Moscovici & Pérez, 1997; Pérez et al., 2001). Starting from the social representations theory (SRT) (Moscovici, 1961/2015, 1984; Wagner, 1996), Pérez et al. (2007) studied the way in which members of an ethnic minority, i.e. the Roma people in Spain, were grouped and driven outside the realm of humanity, to be located closer to the animal kingdom. Their main focus was on the attribution of a different “ontology” to outgroup members. In particular, the ontologization process is the use of a social representation implying the nature–culture and animal–human binaries, to classify both ingroup and outgroup members (Pérez et al., 2001).

Social representation theory has defined these dyadic oppositions, which generally involving ego–alter interactions, as proto-themata (Marková, 2007, 2015) or themata (Moscovici & Vignaux, 1994). Proto-themata are “very basic relational categories” (Marková, 2007, p. 170) that “operate [implicitly] at a non-conscious level” in public discourses (Marková, 2015, p. 4.9). Only when proto-themata explicitly enter public discourse and debate they become “themata” and begin to generate and transform social representations (Marková, 2015). In the SRT literature, themata are not considered universal for every group/society (see Marková, 2003) but are viewed as antinomies but occasionally also apola-triads (Liu, 2004; Marková, 2000), loaded with meaning that are thematized under certain historical, political, economic and social conditions that generate and transform social representations. In other words, themata are contextually and historically situated.

The history of Western civilization has been characterized by an attempt to distinguish humans and animals by means of dimensions such as rationality, language or consciousness in the belief that these attributes have allowed humans to rise from the irrational, instinctual animal world and to enter the superior domain of culture (Agamben, 2004; Baratay, 2003; Rivera, 2000).

Studies of the ontologization process have mainly focused on the Roma minority, as this group vividly represents the “natural” world and, at the same time, the “animal” world in Western societies. Pérez et al. (2001) suggested that when an ethnic minority constantly withstands the majority’s social integration strategies, the majority attributes the absence of integration to the minority’s different essence and its inability to abandon an animal condition. This animal condition creates a new ontology for the minority members, excluding them from humanity in the minds of the majority. Empirical research across four countries, that is, Spain, Britain, Romania and Italy (Berti et al., 2013; Marcu & Chryssochoou, 2005; Pérez et al., 2001), consistently found that more cultural/human characteristics are attributed to the ingroup than to the Roma outgroup, whereas more natural/animal characteristics are assigned to the Roma outgroup than to the ingroup. In Spain, Pérez et al. (2001) found that Roma were attributed more natural (or animal-like) characteristics when participants were informed that Roma had not socially integrated despite the majority’s efforts to integrate them. Marcu and Chryssochoou (2005) asked British and Romanian participants to rate their national ingroup and the Roma group, using characteristics judged to be typically human and typically animal. The results indicated that an ontologization process had taken place with regard to the Roma for both the British and Romanian samples: more cultural characteristics were attributed to the ingroup than to the Roma (outgroup), whereas more natural characteristics were assigned to the Roma than to the ingroup. The attribution of more animal-like characteristics to the outgroup serves to create a social distance between groups and to exclude Roma from the realm of humanity. Pérez et al. (2007) studied the way in which members of an ethnic minority, in this case the Roma people in Spain, were grouped together and driven outside the realm of humanity, to be located closer to the animal kingdom. They found that the Roma minority underwent a more negative ontologization process and were assigned more animal-like characteristics when the participants were primed with a picture of a wild animal representing nature (e.g. a monkey) as compared to when they were primed with a picture of a domestic animal representing human culture (e.g. a dog). In the case of a wild animal representations, the presence of untamed nature acts as a threat against culture (i.e. the human being), challenging the anthropological differentiation between humans and animals (i.e. the use of tools and the development of language and culture). However, according to Pérez et al. (2007), in the case of the Roma minority, the ontologization process seems to operate in order to reduce this threat by locating them closer to the animal pole of the human–animal continuum as the attribution of non-human characteristics that majorities tend to attach to the Roma is functional in terms of outgroup distancing and is associated with the perception of threat that this outgroup exerts on the cultural component of the ingroup. In effect, the cultural component attributed to the ingroup is the foundation of its social identity and representation. In turn, the denial of humanity with regard to the outgroup is a good predictor of discriminatory behaviours with respect to majorities (Orosz et al., 2018).

## Roma People Are Dirty, Thieves and Nomads: Current Prejudices with Ancient Roots

Roma people, one of the largest minority groups in Europe (Piasere, 2012), are a constellation of various cultural groups who have always borne the brunt of discrimination. If those currently living in Europe joined together to create a “Roma nation”, they would count more citizens than in some of the other European countries. The members of this “large minority” are spread out throughout Europe, either as migrants or as commuters, and in many countries, they come up against prejudice and social exclusion (Kende et al., 2021; Lášticová & Findor, 2016; Ljujic et al., 2013; Orosz et al., 2018). Indeed, Roma are generally perceived as criminals and a group of people that threatens the economic and cultural resources of the host country (Kende et al., 2017; Ljujic et al., 2013). Widespread anti-Roma prejudice is often expressed in explicit ways in public discourse, in the media, in policy decisions and in institutional practices.

Roma people have lived in Italy since the fifteenth century. Nevertheless, in the 1970s, the Roma people from Eastern Europe (especially from the countries of the former Yugoslavia and Bulgaria and Romania) started arriving in large numbers. According to a study by the Italian 21st July Association (www.21luglio.org) and estimates by the Council of Europe (2012), in 2019, the presence of Roma, Sinti and Caminanti in Italy ranged between 120,000 and 180,000 people (approximately 0.23% of the population). With reference to anti-Roma prejudice, the studies that have been carried out in an Italian context (e.g. Fontanella et al., 2016; Meneghini, 2017) revealed that this prejudice has ancient roots and it does not tend to diminish over time but, on the contrary, becomes increasingly obstinate and resistant with accompanying acts of frequently committed discrimination (cf. anti-ziganism: Piasere, 2012).

The Roma people have always been portrayed as nomadic, criminal, deceitful, dangerous, dirty people. Indeed, Italians tend to attribute to them a stereotype accompanied by feelings of contempt (Villano et al., 2017; Volpato & Durante, 2008), and comparisons in terms of Italians’ attitudes and level of social acceptance towards various derogated ethnic groups revealed more negative attitudes to the Roma than to others (Meneghini & Fattori, 2016). In addition, the desired social distance with respect to the members of the Roma group is greater. On the other hand, Italians perceive Roma as people who are reluctant to integrate. The consequence of this is that the differences between the two groups (i.e. the Italians and the Roma) are highlighted and a kind of justification for the social exclusion of the outgroup (the Roma), and the need for control by the majority group (the Italians) is provided. Indeed, Meneghini (2017) suggested that in the attitude of the Italians towards the integration of the Roma people, two opposite tendencies operate. On the one hand, in the public sphere, there is a strong demand for assimilation, i.e. a greater adaptation of Roma people to the characteristics of Italian society (Piasere, 1999). On the other hand, in the private sphere, Roma people are “granted” the opportunity to maintain their cultural characteristics (Costarelli, 1994), as this serves to underline the differences between Italians and Roma and to justify the need for control by the majority. An example is the phenomenon of nomadism. As mentioned above, Italians have an image of Roma as being nomadic people. According to Costarelli (1994), the Roma themselves do not seem to oppose this image, so much so that they end up representing themselves and behaving like nomads. In turn, this favours mutual social closure and the consequent vision of the Roma people as an “isolated social atom” (Galli et al., 2007, p. 13) who refuse and to whom integration is refused. To sum up, it could be argued that the Roma themselves are to blame for the rejection by the Italians: if the Roma people were a little less Roma, it would make them more acceptable (Piasere, 1999). The consequent lack of direct contact between the two groups, which seems to favour subtle rather than expressed prejudice towards the Roma (Kende et al., 2017), may be the cause and the effect of the Italians’ prejudice against Roma and may enhance feelings of threat in the majority. On the part of Roma, the desired social distance expressed by Italians may foster detachment from the majority group. As a consequence, a vicious circle, in which social distance and prejudice are self-feeding, may be hypothesized.

### “The Chinese Never Die”

The first Chinese communities settled in Milan as early as 1920, and to this day, their presence has grown slowly but steadily. Most of the Chinese immigrants present in Italy originate from the Chinese province of Zhejiang, and unlike other migrations, it is characterized by family migration.

According to Istat data, there are 290,681 Chinese people living in Italy (Istat, 2018), representing the fourth largest ethnic group. They are frequently defined as being an “impenetrable and closed” group, and Ruvolo (1999) stated that the arrival of the Chinese in Italy generated a certain amount of discontent as they are seen as “those who take away our jobs” (p. 67). As La Barbera and Cariota Ferrara (2010) found, the Chinese are often perceived to be skilled but unfair competitors from an economic point of view and that they are very different to Italians in terms of culture and traditions. In fact, if initially (i.e. in the 1990s) the Chinese immigrants were employed as subcontractors, especially in the field of manufacturing, over time they began to replace the local entrepreneurs, buying their own businesses (including many shops and restaurants) and changing the face of the local neighbourhoods in Italian cities.

The difficult economic situation that already characterized Italian society before the COVID emergency has resulted in Italians blatantly expressing negative attitudes towards the Chinese who represent competition. They are discriminated against by means of a variety of stereotypes and generalizations such as “the Chinese are everywhere”; “they work illegally without labour contracts”; and “the Chinese never die”. Chinese immigrants are invariably seen as being “culturally diverse” from Europeans, due to the fact that their language, writing and customs are different and they appear to resist acculturation and assimilation within the host society. Many Italians think that they tend to isolate themselves from the majority by settling and working in specific neighbourhoods (i.e. the “Chinatowns” in Milan and Rome) as a sign of their unwillingness to integrate (La Barbera & Cariota Ferrara, 2010).

A review of Asian American stereotypes demonstrated that Asians tend to be characterized by Americans as being competent but unsociable (Lin et al., 2005), and in the classic study carried out by Katz and Braly (1933), the Chinese in America were seen as clever (competent) but also conservative, tradition-loving, superstitious and loyal to their families. In a later replication of the Princeton Katz-Braly paradigm, the Chinese were seen as intelligent, industrious and scientifically minded, but still reserved and loyal to family ties (Leslie et al., 2001). Seeing others as lacking traits related to the warmth dimension may imply denying other more positive traits such as honesty, sincerity, sociability and emotional sensitivity. According to Martínez et al. (2012), this denial may lead to others being seen, for example, as robot-like.

Some studies conducted in USA and Australia have shown that, according to Haslam’s model of dehumanization (Haslam, 2006), the Asians generally—and the Chinese particularly—were regarded as highly intelligent (Madon et al., 2001), an aspect of higher cognition associated with HU. When the Chinese directly compared their group with Australians, they viewed their ingroup as more sophisticated (Kashima et al., 2003), reflecting a higher HU aspect of refinement. Thus, it seems that, as compared to Australians, the profile of the Chinese is likely to involve relatively high HU and low HN (Bain et al., 2009).

Moreover, a series of studies carried out by Kteily et al. (2015) on blatant dehumanization has shown that Chinese people are inferior on the “Ascent of Man” scale (with five silhouettes depicting the physiological and cultural evolution of humans, from early human times to the present). Participants (study 1) reported that some groups were less evolved than Americans, and the amount of dehumanization varied across groups: for example, participants blatantly dehumanized Chinese people, South Koreans, Mexican immigrants and, particularly, Muslims and Arabs.

Although there are many studies on the dehumanization process, there are still few studies in the literature concerning the Chinese group in particular, especially within the Italian context. For this reason, we selected Chinese people as one of the targets of our research.

## Overview and Aims of the Study

In the study of outgroup discrimination, the ontologization and the dehumanization approaches have moved along parallel tracks. In our opinion, this is rooted in the traditional distinction in social psychology between the “minoritarian” SR approach, developed in south-Europe, such as France, Spain and Italy, and the mainstream social cognition approach, typical of the Anglosphere (Jost & Kruglanski, 2002). While the SRT specifically derives from the work of Serge Moscovici (1961/2015), the broader SR approach entails a general theoretical orientations which is rooted in Moscovici’s work and embraces the study of the role of structural and ideological factors in the framing of human behaviour (Elcheroth et al., 2011). In this paper, our theoretical inclination is more towards a broader SR approach over specific affiliations.

We believe that some attempts can be made to reconcile the two approaches, namely, dehumanization and ontologization, based on the following reasoning. The two approaches are close in the sense that they both describe a process of denying humanity to social groups based on the distinction between nature and culture. They both share the idea that outgroup members are more similar to animals than ingroup members. Both approaches contrast the ingroup humanity with the outgroup lack of humanity, and both rely on the human–animal dichotomy (Marcu, 2007; Tileaga, 2007). According to the metaphor-based approach, outgroup members are assimilated to animals, due to the lack of HU traits. In our view, this idea is similar to the asymmetric attribution of animal traits to minority members, within the ontologization approach. Secondly, at the empirical level, animalistic dehumanization and ontologization have been similarly investigated via the attribution of human and animal traits to the ingroup and the outgroup. In some cases, the very same stimulus word was used to measure animalistic dehumanization and ontologization. For instance, Saminaden et al. (2010) used stimuli words such as polite, analytic, impulsive and simple, whereas Berti et al. (2013) used educated, instinctive and simple. As for the difference between them, the research within the animalistic dehumanization involved either explicit or implicit measures, while research within the ontologization approach involved only explicit measures.

As per the definition, the ontologization approach consists in the attribution of animal characteristics, besides Pivetti et al. (2018), to our knowledge, no studies have been conducted to investigate the attribution of a different ontology to outgroup members in terms of automata. Pivetti et al. (2018) has proposed a theoretical development of the ontologization approach, which also includes the attribution to outgroup members of a “robot” essence, in addition to the animal essence. This study confirmed the animalization of the Roma people in Italy resulting from ingroup members attributing animal-like associates to them and denying human-like associates. However, the Chinese were ontologized based on a mechanistic approach and were attributed characteristics according to a more automata-like dimension (as compared to animal or human dimensions). Along these lines, we aim to propose a theoretical development of the SR approach by including a process of mechanization of certain outgroups. Outgroup members can be assigned a different ontology, namely, a robot-like essence, as part of an ontologization process. As animalization is the process of anchoring human being to the animality, then mechanization can be seen as a parallel process of anchoring to an automata ontology. Generally speaking, mechanization is the process of changing from working largely or exclusively by hand or with animals to doing that work with machinery and is often related to industrialization and the replacement of human labour. Mechanization conveys the idea that thought is a form of computation shared by human brains and by a class of computing machines and that fundamental elements of mental life, such as intentionality, could be understood on the basis of a physical law. Those elements have laid the foundations of cognitive science and artificial intelligence (Dupuy, 2001). However, no study to date has investigated the multidimensional nature of the ontologizing process for the Roma and Chinese target groups via structural equation modelling (SEM), and no standardized measurement of the ontologizing process with regard to the three mentioned essences (i.e. human, animal and robot) is available.

This study aims to bridge these gaps. This study aims to make an attempt to integrate the two approaches, namely, dehumanization and ontologization, and to put forward a theoretical development of the SR approach, by including the attribution of automata (besides human and animal) essence to ingroup and outgroup members. In a first step, we propose a preliminary contribution to the multidimensional validation of an instrument measuring the attribution of animal, human and robotic essences to the outgroups and to the ingroup. As a second step, we compare three target groups (Roma, Chinese and Italian) with respect to the attributions of animal, human and robotic essences, assuming that Roma are assigned more animal associates, while the Chinese are assigned more robot-related characteristics.

## Methods

### Participants

There were 421 participants in the study, all of whom Italian. Three hundred and fifteen were female (74.8%; missing = 1.2%), and ages ranged from 18 to 83 (M = 29.8, DS = 12.03). Two hundred and ten participants were students (49.9%), 163 were employed (38.7%), 24 were unemployed (5.7%), 13 were worker–student (3.1%), 9 were retired (2.1%), and 2 were housewives (0.5%). With regard to their educational standard, the sample was made up mostly of highly qualified people: 193 had at least completed secondary school (45.8%); 123 had graduated from university (29.2%); and 81 had a post-graduate degree or doctoral qualification (19.2%). The remaining participants had a lower secondary school diploma. Concerning political orientation, 228 positioned themselves to the left of a left–right axis (54.1%), 86 positioned themselves in the centre (20.4%), and 107 positioned themselves to the right (25.4%).

The participants could access the questionnaire on Google Forms. The survey was open for 33 weeks from September 15, 2017, to May 8, 2018. As the target group of the questionnaire included Chinese people, it should be noted that the research was carried out in the pre-COVID-19 era. The participants were recruited from among undergraduate students by means of a convenience sampling strategy (Etikan et al., 2016; Kerlinger, 1986) which included the contacts of the researchers and the research assistants. “Snowball” procedures were also used (Biernacki & Waldorf, 1981). In order to ensure heterogeneity, the questionnaire link was posted by research assistants on social networks or sent to mailing lists including both close friends and contacts and more distant acquaintances. Prospective participants were asked to fill in a questionnaire “on social perceptions”. No fee was provided for participation.

The questionnaire took 25 min to fill in on average. The research complied with the WMA-Declaration of Helsinki (1964/2013) and with the Code of Ethics of the Associations of Italian Psychologist (AIP) (2015). The research received the approval of the Bioethical Committee of the University of Bologna.

## Measures and Procedure

A within-subject design was used to investigate the attribution of human, animal and automata-like associates to three target groups: “typical Roma”, “typical Chinese” and “typical Italian”. The order of presentation of the three target groups was randomized.

With the aim of helping participants to focus on the specific target group in question, before starting the task for each group, they were asked to describe a “typical Roma” vs a “typical Chinese” vs a “typical Italian” person in their own words by means of a free-association task (Bonomo et al., 2013; de Rosa, 2003; Deschamps, 2003).^1^

The participants rated the each of the three target group (“Roma”, “Chinese” and “Italian”) using a set of 24 randomly ordered associates (see Tab. 1). They were requested to indicate to what extent three positive human associates (e.g. intelligent), six negative human associates (e.g. envious), three positive animal associates (e.g. simple), three negative animal associates (e.g. uncontrollable), three positive automata associates (e.g. efficient) and three negative automata associates (e.g. dependent) describe a typical member of the “target” group (range, 1 = not at all to 7 = a great deal). The human, animal and automata associates had been selected on the grounds of previous studies (Berti et al., 2013; Pivetti et al., 2018).

**Table 1:** List of animal/human/automata positive/negative associates

Animal positive associates	Animal negative associates	Human positive associates	Human negative associates	Automata positive associates	Automata negative associates
Simple Sociable Adaptable	Uncontrollable Wild Noisy Instinctive Aggressive Free	Intelligent Rational Educated	Envious Immoral Cruel Selfish False Rapacious	Efficient Technological Active	Dependent Insensitive Passive

The socio-demographic items relating to the participants, such as gender, age, standard of education (primary, lower secondary, upper secondary and university level), occupation and political orientation, (left, centre-left, centre, centre-right, right wing) were included in the last section of the questionnaire (Table 1).

## Results

### Preliminary Analysis

The ratings for the positive and negative human/animal/automata associates were averaged into six mean scores for each target group. Descriptive statistics, reliabilities and correlations between the study variables are reported in Table 2 for the Roma target, in Table 3 for the Chinese target and in Table 4 for the Italian target (i.e. the ingroup).

**Table 2:** Descriptive statistics, reliabilities and correlations of the study variables for the Roma target

	1	2	3	4	5	6
Animal positive index	-					
Animal negative index	.18**	-				
Human positive index	.42**	.05	-			
Human negative index	−.04	.74**	−.11*	-		
Automata positive index	.39**	.25**	.60**	.13**	-	
Automata negative index	.11*	.48**	.01	.67**	.10*	-
N. of items	3	6	3	6	3	3
Mean	3.59	4.96	2.83	3.96	3.01	3.21
SD	1.34	1.44	1.10	1.63	1.19	1.33
Ordinal Cronbach’s a*lpha*	.57	.89	.64	.91	.56	.57
Ordinal *ωt*	.57	.89	.65	.92	.59	.58
Ordinal *ωh*	.57	.88	.65	.91	.59	.58

^**^p ≤ .01; *p ≤ .05

**Table 3:** Descriptive statistics, reliabilities and correlations of the study variables for the Chinese target

	1	2	3	4	5	6
Animal positive index	-					
Animal negative index	.35**	-				
Human positive index	.53**	.22**	-			
Human negative index	.22**	.55**	.17**	-		
Automata positive index	.57**	.29**	.73**	.19**	-	
Automata negative index	.35**	.32**	.29**	.61**	.35**	-
N. of items	3	6	3	6	3	3
Mean	3.87	2.44	4.76	2.72	5.45	3.56
SD	1.26	.93	1.39	1.15	1.37	1.29
Ordinal Cronbach’s a*lpha*	.55	.78	.82	.87	.81	.58
Ordinal *ωt*	.56	.79	.82	.87	.81	.58
Ordinal *ωh*	.55	.79	.82	.87	.81	.58

^**^p ≤ .01; *p ≤ .05

**Table 4:** Descriptive statistics, reliabilities and correlations of the study variables for the Italian target

	1	2	3	4	5	6
Animal positive index	-					
Animal negative index	.58**	-				
Human positive index	.63**	.41**	-			
Human negative index	.30**	.60**	.19**	-		
Automata positive index	.60**	.45**	.79**	.24**	-	
Automata negative index	.29**	.47**	.18**	.72**	.19**	-
N. of items	3	6	3	6	3	3
Mean	4.42	3.92	4.18	3.55	4.12	3.48
SD	1.22	1.08	1.24	1.30	1.24	1.18
Ordinal Cronbach’s a*lpha*	.65	.76	.80	.88	.73	.64
Ordinal ωt	.67	.77	.82	.88	.74	.65
Ordinal *ωh*	.67	.77	.83	.88	.74	.64

^**^p ≤ .01

Referring to the work of Crutzen and Peters (2017) and Peters (2014) with regard to the reliability of ordinal scales, ordinal Cronbach’s *alpha* was computed (Gadermann et al., 2012), in addition to the ordinal *omega* (total) (*ωt*) (McDonald, 1978) and the ordinal *omega* (hierachical) (*ωh*) (McDonald, 1999). This was done by means of a polychoric correlation matrix using the “userfriendlyscience” (Peters, 2015) the source package R (R Development Core Team, 2014).

With regard to the Roma target group, all but three of the variables were intercorrelated: (1) the negative animal index and the positive human index, (2) the positive animal index and the negative human index and (3) the negative automata index and the positive Human index. Cronbach’s *alpha* revealed a good internal consistency for two indexes (i.e. the negative animal and negative human indexes) and was approaching an acceptable threshold in all the other cases.

The results for the Chinese target group showed that the variables were all highly positively intercorrelated. Cronbach’s *alpha* computations showed good internal consistency for four indexes, with the exception of the positive animal and negative automata indexes which approached an acceptable threshold.

For the Italian target group, the variables were all highly positively intercorrelated. Cronbach’s *alpha* computations showed good internal consistency, with scores above the 0.64 threshold.

## Confirmatory Factor Analysis

**Fig. 1:**
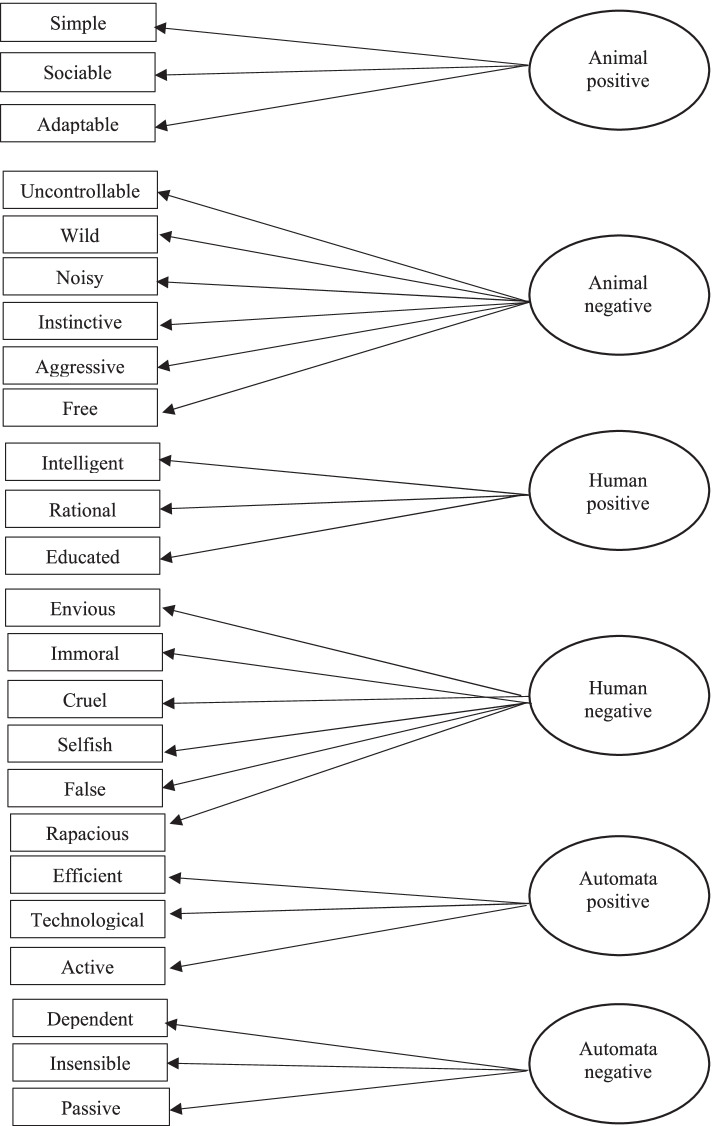
Confirmatory factor analysis (CFA): hypothesized model

The same confirmatory factor analysis (CFA) model was used to test each of the three target groups (i.e. Roma, Chinese and Italian). LISREL 8.80 software was used (Jöreskog & Sörbom, 1988, 1996, 2001). The model included six latent variables (i.e. positive and negative animal, positive and negative human and positive and negative automata index) and 24 observed variables (24 items relating to the 6 latent variables). In the hypothesized model (see Fig. 1), the 24 observed variables pointed to the 6 latent variables according to the scheme indicated in Table 1.

As the observed variables were an ordinal scale (Likert), the polychoric correlation matrix was used as input data, weighted by the asymptotic covariance matrix. Given that the multivariate normality in the observed variables was not satisfied, we used the robust unweighted least squares (*RULS*) as the method of estimation (Morata Ramirez, & Holgado-Tello, 2013). As for the model fit index, we considered *RMSEA*, *NFI*, *NNFI* and *CFI* (Hu & Bentler, 1999).

The procedure was carried out in two separate steps. In the first step, we tested the hypothesized full LISREL model (model 1, Fig. 1). As the fit indexes obtained in this way were not considered sufficiently satisfactory, the model was improved by correlating the error variables of some items. This operation is generally allowed when the pairs of error variables imply the presence of latent variables (not included in the model). Specifically, we formed the pairs “uncontrollable and aggressive”, “free and instinctive” and “uncontrollable and wild” because they belong to the same factor (animal negative), and this makes the operation permissible. We then formed the pair between the items “active and passive” because they constitute a well-known dichotomy, which could represent the latent variable not included in the model. We finally formed the last three pairs by virtue of the fact that the corresponding variables normally belong to the same semantic area and that the respondents, as naïve scientists, formulated their answers based on this common sense: (a) the pair “cruel and aggressive” refers to the common-sense idea that an aggressive man or animal can also be considered cruel; (b) the pair “educated and technological” brings to mind the man/machine relationship (e.g. personal computer, automata) seen as a privilege of a few people who possess the right knowledge necessary to use the machines competently; and (c) the pair “false and insensitive” was then created because in the Italian language, these are polysemic terms that can be coupled both in reference to automata (“the robots mimicking human behaviour will never be perfect ‘humans’” (Lakatos et al., 2014, p. 3/32) because they are perceived as artificial and insensitive to emotions) and referring to human beings, such as in common meaning of the figure of the conman (who can achieve their goals because he/she is false and insensitive, not empathetic). In order to make Fig. 1 clearer, the arrows showing the correlations between *KSI* variables (*phi matrix*) were omitted.

For each of the three target groups, the second model obtained in this way (model 2) indicated significant decreases in ${\chi }^{2}$ for each of the groups (*p* < 0.000 with *ΔT* = 153.5 and *Δdf* = 7 for the Roma; *p* < 0.000 with *ΔT* = 190.2 and *Δdf* = 7 for the Chinese; *p* < 0.000 with *ΔT* = 196.2 and *Δdf* = 7 for the Italians), and satisfactory goodness-of-fit statistics with Satorra-Bentler scaled ${\chi }^{2}$ (230; *n* = 421) ranged between 590.69 and 710.69. Other fit indexes were acceptable: *RMSEA* ranged between 0.06 and 0.07, *NFI* ranged between 0.95 and 0.96, and *NNFI* ranged between 0.96 and 0.97 and *CFI* = 0.97 in all three target groups, following Schermelleh-Engel et al. (2003). The fit indexes are reported in Table 5. Model 2 was retained, given that ${\chi }^{2}$ decreased, and the fit indexes for Model 2 were an improvement on model 1.

**Table 5:** Confirmatory factor analysis for the proposed models for the each of the three targets (Roma, Chinese and Italian): goodness-of-fit indexes

Targets		Satorra–Bentler scaled c*hi*^*2*^ (*n* = 421)	*ΔT, p (Δdf* = *7)*	*RMSEA*	*NFI*	*NNFI*	*CFI*
Roma	Model 1:	795.62 (*df* = 237)	ΔT = 153.5, *p* < .000	.08	.90	.91	.92
	Model 2:	642.12 (*df* = 230)		.07	.96	.97	.97
Chinese	Model 1:	780.87 (*df* = 237)	ΔT = 190.2, *p* < .000	.07	.89	.89	.90
	Model 2:	590.69 (*df* = 230)		.06	.95	.96	.97
Italian	Model 1:	906.89 (*df* = 237)	ΔT = 196.2, *p* < .000	.08	.90	.90	.92
	Model 2:	710.69 (*df* = 230)		.07	.95	.96	.97

Table 6 shows the standardized solutions (*lambda* × *matrix*) for the three target groups. The cut-off value was arbitrarily selected on the basis of the area of study, but ± 0.4 seems to be preferred by many researchers (Salkind, 2010). According to a rule of thumb, using an *alpha* level of 0.01 (two-tailed) and a rotated factor loading for a sample size of at least 300, a factor loading would need to be at least 0.32 to be considered statistically meaningful (Tabachnick & Fidell, 2007; Yong & Pearce, 2013). Our sample comprises 421 participants, and factor loadings were all above this threshold, with the exception of the “free” item for the Chinese target group.

**Table 6:** Standardized solutions (*lambda* × *matrix*) for the three targets (Roma, Chinese and Italian)

	Roma	Chinese	Italian
Animal positive			
Simple	.45	.59	.52
Sociable	.75	.44	.82
Adaptable	.45	.62	.64
Animal negative			
Uncontrollable	.78	.62	.51
Wild	.83	.65	.46
Noisy	.71	.59	.75
Instinctive	.67	.57	.73
Aggressive	.92	.85	.72
Free	.49	.15	.34
Human positive			
Intelligent	.66	.90	.83
Rational	.61	.70	.73
Educated	.61	.79	.76
Human negative			
Envious	.64	.66	.78
Immoral	.77	.77	.74
Cruel	.91	.76	.63
Selfish	.89	.79	.81
False	.79	.68	.70
Rapacious	.80	.73	.85
Automata positive			
Efficient	.67	.81	.80
Technological	.49	.81	.63
Active	.56	.74	.69
Automata negative			
Dependent	.34	.50	.59
Insensitive	.91	.71	.69
Passive	.43	.45	.57

## Ontologization Process

In order to investigate the way in which the six indexes were ascribed to the three targets, we carried out a repeated *ANOVA* measure, with the 18 indexes varying within subjects. All effects are reported as significant at *p* < 0.001 unless otherwise stated. *Mauchly’s test* indicated that the assumption of sphericity had been violated, ${\chi }^{2}$ (152) = 2153.1; therefore, *Greenhouse–Geisser* corrected tests are reported (ε = 0.56). The results showed that the 18 variables differed significantly *F*(9.497, 3988.801) = 263.071, *η*_*p*_^*2*^ = 0.39.

Pairwise comparison showed that as for the Roma target, it was attributed with more animal positive than human positive associates and with more animal positive than robot positive associates. This pattern of results was the same as for negative associates, with higher attribution of animal negative than human negative and of animal negative than robot negative associates. In addition, the target was rated as more human negative than human positive and more human negative than robot negative. Those results showed that the Roma target was animalized both on a positive than negative dimension, whereas humanization flowed through a negative evaluation of the target.

The Chinese target was given more robot positive than human positive associates and more robot positive than animal positive associates. This pattern of results was similar for the negative side, with more robot negative than human negative associates and more robot negative than animal negative associates given to the target. Those results indicated that the target was ontologized based on the automata (positive and negative) dimension. Moreover, the target was given more human positive than animal positive associates, more human positive than human negative associates and more human negative than animal negative associates, showing that also the human dimension was used to evaluate the target.

As for the Italian target, it was given more animal positive than human positive and more animal positive than robot positive associates. Also on the negative side, it was described as more animal negative than human negative and more animal negative than robot negative. Taken together, those results indicated that the Italians perceived themselves as more animal (positive or negative) than human or robot. Additionally, the ingroups were attributed more human positive than human negative and more human positive than robot positive associates, showing a positive self-humanization. As for the robot dimension, the target was perceived as more robot positive than robot negative and as human negative as robot negative.

**Fig. 2:**
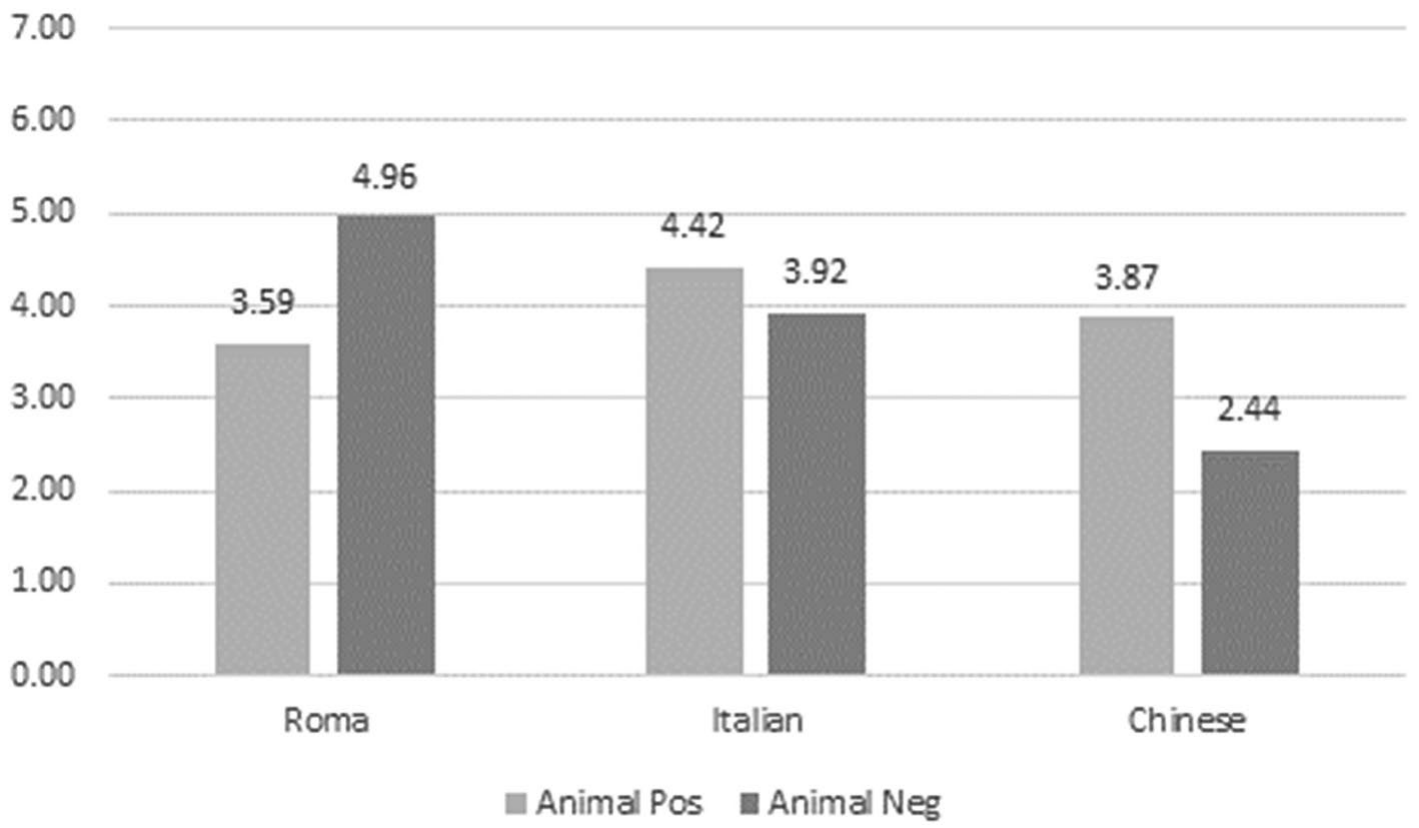
Attribution of animal positive and negative associates to the three targets

As for the attribution of animal positive and negative associates to the three targets, the Roma target was ascribed with more animal negative associates than any other target and with more animal negative than animal positive associates, showing an animalization of the target in terms of negative evaluation. The Italian target was attributed more animal positive than animal negative associates and more animal positive associates than any other target. It was attributed more animal negative associates than the Chinese. The Chinese target was attributed more animal positive than animal negative associates and more animal positive ones than the Roma target. Those results indicate the target was positively evaluated on the animal dimensions (see Fig. 2).

**Fig. 3:**
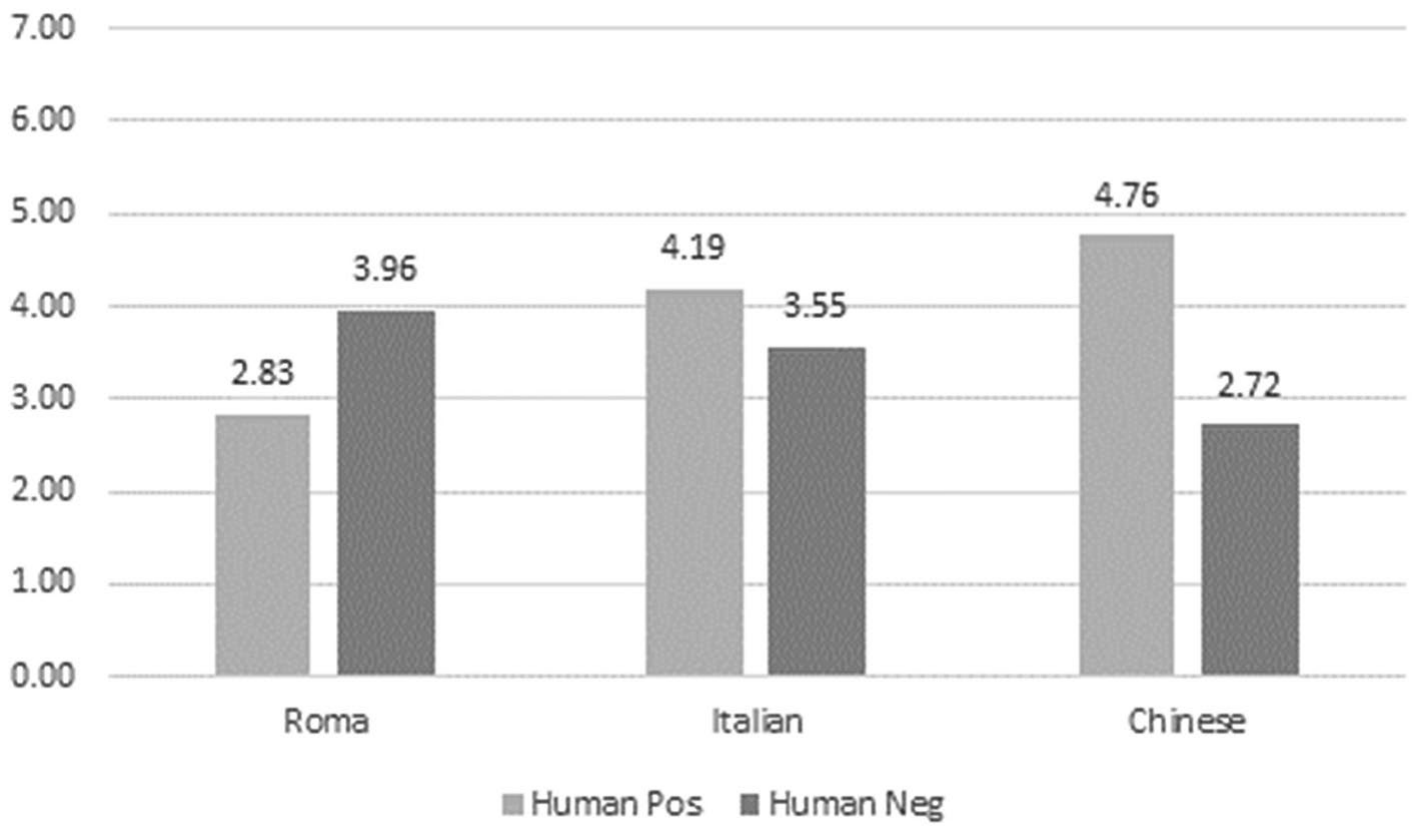
Attribution of human positive and negative associates to the three targets

As for the attribution of human positive and negative associates to the three targets, the Italian target was attributed more human positive associates than the Roma target and more human negative associates than the Chinese. The Roma target was perceived as more human negative than any other target, showing that some albeit negative form of humanity is attributed to the Roma. The Chinese target was rated as more human positive than any other target, showing that a positive form of humanity is attributed to the target (see Fig. 3).

**Fig. 4:**
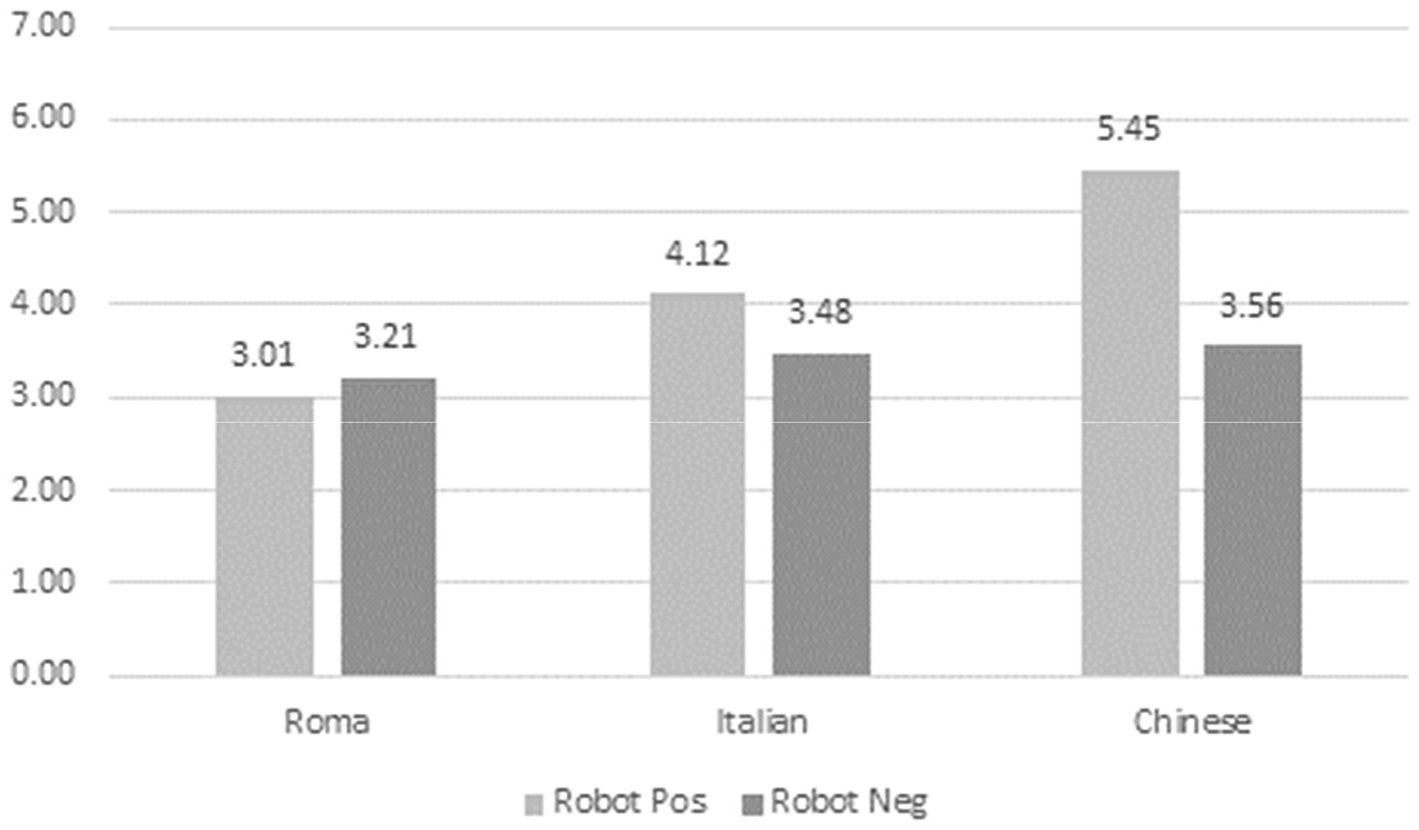
Attribution of robot positive and negative associates to the three targets

As for the attribution of robot positive and negative associates to the three targets, the Chinese target was attributed more robot positive associates than any other target. Also, it was described as more robot negative than the Roma target. Moreover, it was described as being more robot positive than robot negative. Secondly, the Italian target was rated as more robotic than the Roma target, both in terms of positive and negative associates (*p* = 0.029). It was attributed as many robot negative associates as the Chinese. Generally, those results indicated that the Chinese target was ontologized in an automata way, with also a prevailing positive evaluation in terms of robot attribution. The Roma target was rated similarly in terms of positive and negative robots, showing that the robot dimension was not relevant for the evaluation of the Roma target (see Fig. 4).

## Discussion

Ethnic prejudice is considered socially unacceptable, and as a result, certain subtle forms of racism have developed (Pettigrew & Marteens, 1995), which are harder to detect. Acts of discrimination have gradually become more hidden, and widespread prejudice implicitly acts by means of subtle derogatory attributions to the outgroups. This mechanism is embodied in the ontologization process that characterizes the way in which majorities tend to act towards ethnic minorities.

This study is framed around two primary goals. Firstly, under the umbrella of contributing to the understanding of the role of ontologization processes in shaping intergroup relations, this study proposes a preliminary contribution to the multidimensional validation of an instrument measuring the attribution of animal, human and robotic essences to the immigrant outgroups, in this case, specifically Roma and Chinese people, and to the ingroup. Traditionally, studies on the ontologization processes had focused only on the first two dimensions, that is, animal and human essence. Pivetti et al. (2018) have investigated the attribution of three essences, that is, the animal, human and automata one, to the Roma, Romanian and Chinese target, but have not provided any factorial validation of the proposed model. In this study, using CFA, we found that the model including human, animal and automata associates was statistically robust for each of the three target groups. Specifically, the CFA used a six-dimensional structure to capture the distinct aspects of the ontologization process which apply to the Roma, the Chinese and the Italians, that is, animal positive, animal negative, human positive, human negative, automata positive and automata negative dimension. We consider the fact that the model was statistically robust with each of the three target groups as an indication of the strength of our theoretical considerations and as one of the novelties of this study. Hence, the automata dimension has been added to the ontologization model to grasp the mechanization process of the Chinese target group. In this sense, our study has made a preliminary effort to reconcile the ontologization and the dehumanization approaches, by showing the many similarities they share in terms of attribution of animal/human/automata essence to outgroup members.

The findings also support the idea that the selected items are valid tools to capture the nuances of the ontologization process, in terms of either human, animal and robot positive/negative essences. However, internal consistency indexes show a great deal of variability between the target groups. Whereas the internal consistency values for negative human indexes and negative animal indexes (as measured using 6 items each) have a score above acceptability for each of the three targets, the positive animal indexes and the negative automata indexes (measured using 3 items each) are questionable. Future research could increase the number of characteristics involved in those indexes showing questionable consistency values.

The second goal of this study is to compare the attributions of animal, human and robot essences to three target groups, that is, the Roma, Chinese and Italian. The results show that the Roma target was animalized by attributing more animal negative associates than any other target group. This is in line with a previous study conducted in Italy showing the animalization of Roma people by means of the attribution of animal-like associates (Berti et al., 2013) and with data collected in other European countries (e.g. Hungary: see Orosz et al., 2018). Also, the Roma target was attributed a more human negative essence that any other target. Taken together with the super-attribution of animal negative essence, this result confirmed the negative evaluation of the Roma group, as compared with the Chinese immigrant group.

While the empirical studies within the ontologization framework traditionally focused exclusively on the Roma minority, to our knowledge this is the first attempt, besides Pivetti et al. (2018), to investigate whether this process also occurs for other immigrant groups such as the Chinese group. We consider this as the second novelty of the study. Consistently with the prediction, the Chinese target was mainly attributed a robot positive essence rather than an animal essence. This attribution was higher than any other study group, showing that the Chinese people were perceived as efficient and technological. As the Roma people were attributed a different quality, i.e. an animal-like status, the Chinese immigrant can be attributed a different quality, i.e. an automata-like status.

The increased attribution of a positive characteristic to the outgroup (i.e. robot positive essence to Chinese group) can be explained in the light of classic studies on ingroup and outgroup favouritism (Mummendey & Schreiber, 1983, 1984; Mummendey & Simon, 1989), conducted within the framework of social identity theory (Tajfel & Turner, 1979, 1986). These studies have shown that it is possible to favour the outgroup on dimensions considered unimportant to one’s own group because one’s own social identity is not cast into doubt, while the opposite occurs on dimensions relevant to the ingroup because this would entail an unacceptable threat to one’s own social identity.

The attribution of robot essence in the case of Chinese group was also supported by the predictions of the stereotype content model (SCM) (e.g. Lin et al., 2005) body of literature, indicating that Asians are commonly stereotyped as being competent but unsociable, which makes them potential targets of racial prejudice tinged with envy and discomfort. Starting from a different theoretical perspective, many researchers dealing with the dehumanization approach have also proved that some groups are animalistically dehumanized, while others are mechanistically dehumanized (Viki et al., 2006). Specifically, Bain et al. (2009) also found empirical evidence about how the Chinese group was mechanistically dehumanized by denying HN traits to the group.

Apparently, the attribution of more human essence to the Italian ingroup as compared to the immigrant groups was not verified, with the Chinese being perceived as more human positive and the Roma as more human negative than the ingroups. This is in line with the study by Pivetti et al. (2018), showing that Italians were perceived as human as immigrant groups. However, there is a possible explanation for this unexpected result if the ingroup’s evaluations of the human characteristic of their group is analysed in terms of the attributions to the outgroups of more animal and automata associates. In fact, research on the processes of dehumanization and infrahumanization has shown that people, in order to attribute greater humanity to the ingroup, attribute more animal characteristics (animalistic dehumanization) and mechanistic characteristics (mechanistic dehumanization) to the outgroups. Thus, they indirectly associate more human characteristics to the ingroup and contribute to marking the differentiation between the ingroup and the outgroup (Krueger et al., 2008; Perdue et al., 1990).

Similarly, in this study that simultaneously analysed the attributions of animal, mechanistic and human characteristics within the ontologization perspective, results showed that the participants evaluated the ingroup as “less animal negative than the Roma” and as “less robot positive than the Chinese”. Therefore, the ingroup turned out to have more humanity than Roma and Chinese even if the attributes of “human positive” and “human negative” were not stronger towards the ingroup than towards the outgroups. In other words, considering the human positive and negative alone, the greater humanization of the ingroup did not emerged, but the use of the animalistic and mechanistic dimensions made this tendency visible: the Italian respondents marked the greater humanity of the ingroup by attributing characteristics of “animal negative to the Roma” and of “robot positive to the Chinese”, affirming the differentiation of the ingroup with respect to the outgroups and showing supposed ingroup favouritism bias. Furthermore, these results seem to suggest that the process underlying ingroup favouritism is independent of the positivity or negativity of the associates attributed to the outgroups: it is the presence of these non-human characteristics that implicitly leads to a judgement of the outgroup’s lack of humanity (and consequently of the ingroup’s greater humanity). The primary focus of the process seems to differentiate “us” and “them”, highlighting the incongruity between “our values” and “their values” (Schwartz & Struch, 1989). This also implies that a negative judgement is not necessary to trigger prejudice and rejection of an outgroup. We can hypothesize that the mechanism underlying the attributional process of the participants is similar to the one underlying attitudinal ambivalence bias, i.e. the tendency to experience positive and negative attitudes in the same time towards the same object/person (Bell & Esses, 2002). Ambivalence seems to legitimize prejudice (Costarelli & Gerłowska, 2015). In fact, research has shown that, with the spread of social norms condemning blatant negative attitude towards minorities, people increasingly experience ambivalence in intergroup relations (Has et al., 1992). Attributing less humanity to the outgroup, by means of animal and mechanistic attributes (positive and/or negative), will be enough to see the Roma and the Chinese as people who deserve less than Italians and, as ambivalence influences behavioural intentions (Costarelli & Colloca, 2004), as people who do not deserve help (Andrighetto et al., 2014). According to this, the assessments of the ontologization process towards a specific outgroup may predict how the members of the majority group (the ingroup) behave towards the members of the minority group (López-Rodríguez et al., 2016, 2017).

An additional explanation for the lack of attribution of more human essence to the ingroup may rely on the ingroup-stereotype shared among Italians. According to old adage, Italians are a people of poets, sailors and lovers. For instance, many Italians are described as warm and sociable but also wild and unpredictable, by their northern, European counterparts. Also, the content of the ingroup-stereotype of Italians does not involve the idea of being more cruel and/or more intelligent than immigrant groups. Right-wing political discourse, for instance, refers to the idea that Italians have greater rights to access the jobs market and public houses than immigrant groups based on Italian heritage, no matter how intelligent/capable/in need the immigrant may be.

These hypothesized explanations can be investigated in future research on the ontologization process via the list of associates tested in our model, together with other validated measures (e.g. the outgroup threat). Moreover, this measure could be proficiently used to study prejudice towards other minorities such as LGBT + minority and same-sex couples (e.g. Di Battista et al., 2020) and those social groups/categories that suffer from process of mechanization. The introduction of the automata dimension in the assessment of the ontologization of the outgroups can highlight also these facets of the modern social prejudices.

## Study Limitations

As we resorted to convenience sampling, we are aware that our sample was not necessarily representative of Italians in general. To ensure heterogeneity, candidates were approached across a broad spectrum relating to the study topic. Moreover, the questionnaire was rather long (42 items), and as such, some subjects did not complete it all the way through, but the method of administration via Google Forms allowed us to easily identify and eliminate them from the sample.

As we adopted the theoretical perspective of SR, another limitation of our study would be that by using a questionnaire that surveys human, animal and automaton associates to explore group attributes, we risk treating social representations as a static entity, overlooking a fundamental feature: they are generated by dynamic meaning-making processes present in interaction between and within groups (Wagner, 1996) and transform over time as a result of historical, cultural, social, etc. changes. In this light, it would be interesting to direct our future research into the SR of Roma and Chinese people via longitudinal studies using quali-quantitative methodologies, including, for example, a free-association task in addition to the questionnaire presented in this paper. This would take into account the transformative characteristics of social representations. It would be interesting to see how, following the COVID-19 pandemic (with China as the first hotspot of the illness), the human, animal and automaton associates attributed to the Chinese people have been transformed and, above all, whether new ones emerge.

Moreover, as the themata are contextually situated, in our paper, we presume that human/animal/automata dimensions universally underlie the ontology of majority–minority groups in Western thought. However, we cannot rule out the possibility that there could also be different binaries (or themata) constituting the attributions of groups in non-Western societies. As we start from the SR approach, we are interested in common-sense knowledge, that is, in the lay understanding of intergroup relations. Social representations are theories about the world, socially constructed during everyday conversation, and, at the same time, they are individual mental content about socially relevant phenomena (Wagner, 1994). When people interact through gossip, argue, discuss different issues, read newspapers and watch TV, they are building shared pictures of the world. In this sense, social representations are intrinsic to everyday conversation (Moscovici, 1984). As a consequence, our view is anthropocentric and Western oriented by definition, as the focus of our paper was to investigate the attribution of human/animal/automata dimensions from the point of view of the Western laymen.

## Conclusion

As “[…] prejudice is a collaborative and social relationship-driven endeavor” (Dixon & Levine, 2012 cited in Lášticová, & Findor, 2016, p, 237), a tool to assess ontologization attributions is particularly useful in terms of tracing the profiles of intergroup relations between specific groups, embedded in specific contexts (Patel et al., 2017). This means that people’s attitudes and reactions to an outgroup are shaped (in particular with reference to the relationship between majority and minority groups) both by historical and social factors and by the specific characteristics of the groups in question and the perception that the members of one group have of members of the other group. As a consequence, the features which characterize the relationship between two particular groups cannot be generalized to other groups and other contexts. Investigating the representation of a majority group with regard to each minority group present in their area would help to clarify the motivations underlying prejudices and certain types of behaviours and make it possible to plan targeted interventions aimed at reducing any negative outcomes.

## Data Availability

Data are available from the authors upon request.

## Footnotes

1 Qualitative data emerging from free-association tasks are not presented in this paper.
